# Development and validation of an online model to predict critical COVID-19 with immune-inflammatory parameters

**DOI:** 10.1186/s40560-021-00531-1

**Published:** 2021-02-18

**Authors:** Yue Gao, Lingxi Chen, Jianhua Chi, Shaoqing Zeng, Xikang Feng, Huayi Li, Dan Liu, Xinxia Feng, Siyuan Wang, Ya Wang, Ruidi Yu, Yuan Yuan, Sen Xu, Chunrui Li, Wei Zhang, Shuaicheng Li, Qinglei Gao

**Affiliations:** 1 National Medical Center for Major Public Health Events, Tongji Hospital, Tongji Medical College, Huazhong University of Science and Technology, Wuhan, 430000 China; 2 Cancer Biology Research Center (Key Laboratory of Chinese Ministry of Education), Tongji Hospital, Tongji Medical College, Huazhong University of Science and Technology, 1095 Jiefang Ave, Wuhan, 430000 China; 3 Department of Gynecology and Obstetrics, Tongji Hospital, Tongji Medical College, Huazhong University of Science and Technology, Wuhan, People’s Republic of China; 4 Department of Computer Science, City University of Hong Kong, Tatchee Avenue, Kowloon Tong, 999077 Hong Kong; 5 School of Software, Northwestern Polytechnical University, Xi’an, People’s Republic of China; 6 Department of Gastroenterology, Tongji Hospital, Tongji Medical College, Huazhong University of Science and Technology, Wuhan, 430000 People’s Republic of China; 7 Department of Hematology, Tongji Hospital, Tongji Medical College, Huazhong University of Science and Technology, Wuhan, People’s Republic of China

**Keywords:** COVID-19, Critical illness, Machine learning, Immune-inflammatory parameters, Online model

## Abstract

**Background:**

Immune and inflammatory dysfunction was reported to underpin critical COVID-19(coronavirus disease 2019). We aim to develop a machine learning model that enables accurate prediction of critical COVID-19 using immune-inflammatory features at admission.

**Methods:**

We retrospectively collected 2076 consecutive COVID-19 patients with definite outcomes (discharge or death) between January 27, 2020 and March 30, 2020 from two hospitals in China. Critical illness was defined as admission to intensive care unit, receiving invasive ventilation, or death. Least Absolute Shrinkage and Selection Operator (LASSO) was applied for feature selection. Five machine learning algorithms, including Logistic Regression (LR), Support Vector Machine (SVM), Gradient Boosted Decision Tree (GBDT), K-Nearest Neighbor (KNN), and Neural Network (NN) were built in a training dataset, and assessed in an internal validation dataset and an external validation dataset.

**Results:**

Six features (procalcitonin, [T + B + NK cell] count, interleukin 6, C reactive protein, interleukin 2 receptor, T-helper lymphocyte/T-suppressor lymphocyte) were finally used for model development. Five models displayed varying but all promising predictive performance. Notably, the ensemble model, SPMCIIP (severity prediction model for COVID-19 by immune-inflammatory parameters), derived from three contributive algorithms (SVM, GBDT, and NN) achieved the best performance with an area under the curve (AUC) of 0.991 (95% confidence interval [CI] 0.979–1.000) in internal validation cohort and 0.999 (95% CI 0.998–1.000) in external validation cohort to identify patients with critical COVID-19. SPMCIIP could accurately and expeditiously predict the occurrence of critical COVID-19 approximately 20 days in advance.

**Conclusions:**

The developed online prediction model SPMCIIP is hopeful to facilitate intensive monitoring and early intervention of high risk of critical illness in COVID-19 patients.

**Trial registration:**

This study was retrospectively registered in the Chinese Clinical Trial Registry (ChiCTR2000032161).

**Graphical abstracthelper lymphocytve:**

vv

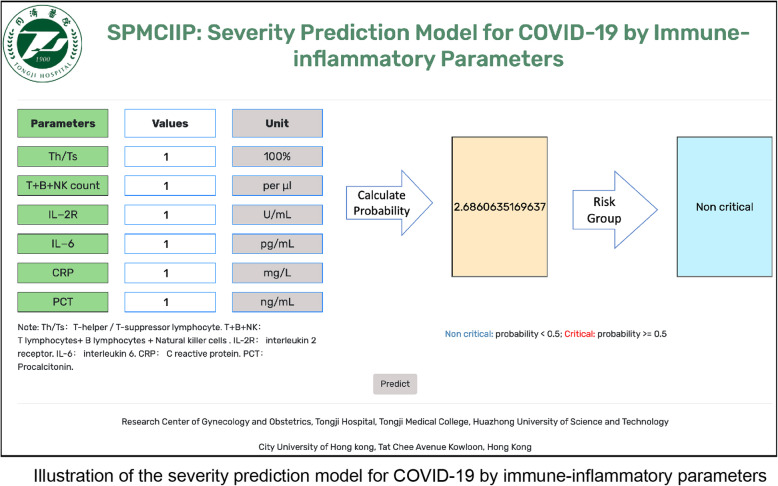

**Supplementary Information:**

The online version contains supplementary material available at 10.1186/s40560-021-00531-1.

## Background

Coronavirus disease 2019 (COVID-19), caused by severe acute respiratory syndrome coronavirus 2 (SARS-CoV-2) [1], ferociously hit the world. Up to September 20, 2020, there had been 30,675,675 confirmed cases and 954,417 deaths worldwide [2]. The reported mortality in critically ill COVID-19 patients was approximately 40%, by contrast with 2.3% for overall patients [3]. Moreover, most patients with critical COVID-19 had relatively mild symptoms prior to physiological deterioration. Therefore, early identification of critically ill patients is crucial for the management of COVID-19.

Immune/inflammatory response of SARS-CoV-2 infection is believed to play an essential role in the progression of COVID-19, though not fully understood [4]. Inflammatory markers, such as C reactive protein (CRP), procalcitonin (PCT), and ferritin, were markedly elevated in critically ill COVID-19 patients [5, 6]. Cytokines play an immunomodulating function, and uncontrolled cytokine storm is responsible for multiorgan dysfunction and poor outcomes of COVID-19 [7]. With both innate and adaptive immune compartments contribution, cytokine storm in COVID-19 is widely concerned [8, 9]. As expected, the differences of multiple cytokines and immune cells between critically ill and non-critically ill patients were observed in clinical practice [4]. Besides, early seroconversion and high antibody (serologic IgM and IgG antibodies against SARS-CoV-2) titer were linked with attenuated clinical symptoms [10].

Immune response of SARS-COV-2 infection is a complex process that has not yet been fully elucidated. Multiple indicators involved may lead to anxiety and confusion of clinicians in patient management. The clinical and imaging features of patients with different disease severity were compared [11, 12], and several prediction models have been established. These prognostic factors mainly included demographic, imaging, and clinical features [13]. Immune-inflammatory parameters have displayed promising prognostic implications, but have not been utilized to enable prediction of critical COVID-19. Traditional methods are not competent in dealing with complex parameters, while machine learning, a sub-discipline of artificial intelligence, may be helpful [14].

The objective of this study is to develop and validate a machine learning model that accurately predicts the occurrence of critical illness in patients with COVID-19 based on immune-inflammatory parameters.

## Methods

### Study design and participants

We conducted a retrospective study that included 2451 consecutive COVID-19 patients with outcomes (discharge or death) between January 27, 2020, and March 30, 2020, from Sino-French New City Campus of Tongji Hospital and Optical Valley Campus of Tongji Hospital in China, who were diagnosed according to the 7th edition of the Diagnosis and Treatment Protocol of COVID-19 by the National Health Commission of the People’s Republic of China [15]. Exclusion criteria were (1) patients under 18 years old, or with pregnancy; (2) patients transferred from Fangcang hospitals for social-distancing; (3) patients died within 24 h of admission, and patients re-hospitalized or discharged for special reasons such as dialysis. Details of excluded patients were as follows: 80 cases without matched diagnosis, 216 cases from Fangcang shelter hospitals, 37 cases died within 24 h, and 42 cases under 18 years old, et al.

As 375 patients were excluded, 2076 patients were finally included in this study and divided into three groups. Specifically, 50% and 50% of patients from Sino-French New City Campus of Tongji Hospital were randomly divided into the training cohort and the internal validation cohort. Patients from the Optical Valley Campus of Tongji Hospital were used as an external validation cohort. Critical illness was defined as admission to intensive care unit, undergoing invasive ventilation, or death [16]. A total of 36 raw immune/inflammatory parameters (natural killer [NK] cells count, NK cell percent, T-helper + T-suppressor lymphocyte [Th + Ts] percent, Th/Ts, Th + Ts count, [T + B + NK] count, [T + B + NK] percent, Th count, Th percent, Ts count, Ts percent, total B count, total B percent, total T lymphocyte-T-helper lymphocyte-T-suppressor lymphocyte [total T-THS], total T count, total T percent, complement 3 [C3], complement 4 [C4], ferritin, lymphocyte [LYM] count, lymphocyte [LYM] percent, C reactive protein, procalcitonin, interferon-γ [IFN-γ], tumor necrosis factor α [TNF-α], interleukin-1β [IL-1β], interleukin-2 receptor [IL-2R], interleukin-4 [IL-4], interleukin-6 [IL-6], interleukin-8 [IL-8], interleukin-10 [IL-10], immunoglobulin A [IgA], immunoglobulin G [IgG], immunoglobulin M [IgM], SARS-CoV-2 specific antibody IgM [C-IgM], and SARS-CoV-2 specific antibody IgG [C-IgG]) were collected from electronic medical records at admission. These features were collected using the same pre-designed data collection table across cohorts. Trained researchers entered and double-checked the data independently.

### Data preprocessing

The medical records contained missing entries (Additional file 2a). To address it, we filtered out patients that harbored more than or equal to 30% missing features, resulting in 222 patients left in Sino-French New City Campus of Tongji Hospital and 228 patients left in the Optical Valley Campus of Tongji Hospital (Additional file 2b). Then, we abandoned the immune-inflammatory parameters missing more than or equal to 30% across the remaining patients, and only 28 features were qualified (Additional file 2c). We utilized the missForest [17] algorithm to estimate the missing entries in the data (Additional file 3). The rationale of choosing 30% as exclusion criteria is to include more patients under the premise of ensuring the imputation robustness. Daniel et al. have demonstrated that missForest can deal with features up to 30% missing values with profound performance [17].

### Feature selection

We first applied LASSO (Least Absolute Shrinkage and Selection Operator) logistic regression to identify the most predictive variables guided by several researches [16, 18]. LASSO utilizes the L1 penalty to make the coefficients of weak features turn to zero during fitting [19]. We regarded features with zero coefficients as redundant, and only non-zero coefficient features were included for model training.

### Model development

We fitted the selected features into five machine learning models, including Logistic Regression (LR), Support Vector Machine (SVM), Gradient Boosted Decision Tree (GBDT), K-Nearest Neighbor (KNN), and Neural Network (NN), to predict patients’ critical illness status with COVID-19. We chose the five models because they are classic models that are representative, widely used in the field of EHR prediction, and sensitive to different data modalities. For instance, based on the decision tree model, GBDT, where features are merely used to split the node, is not sensitive to scale and distribution of features. Scaling or not will not affect the result of the split [20], which also applies to KNN. Therefore, scaling is not required in GBDT and KNN for input training data. LR, SVM, and NN propose models by training weights with the steepest gradient descent algorithm and the steepest gradient ascent algorithm, respectively. They are sensitive to feature scale, so standardizing data is needed to eliminate the differences between features and speed up model convergence [21]. Patients with predictive probability larger or equal to 0.5 are considered high risk, otherwise low risk. To build the ensemble model, we tried different combination of baseline models and found that the combination of SVM, GBDT, and NN with respective weighted voting 0.3, 0.5, and 0.2 achieved the highest AUC. R library “caret” was utilized for model training and prediction with tenfold cross-validation. The LR, SVM, GBDT, KNN, and NN were called with method “glm,” “svmLinearWeights,” “gbm,” “knn,” and “avNNet” with default settings, respectively. Data were scaled and centered before training and testing.

### Statistical analysis

All statistical analysis was performed with R (version 3.6.2). The receiver operating characteristics (ROC) curve and the area under the curve (AUC) analysis were conducted with R “pROC” package. The calibration curve was depicted with R “rms” package. Accuracy (ACC), sensitivity (SE), specificity (SP), positive predictive value (PPV), negative predictive value (NPV), Cohen’s kappa coefficient (Kappa), F1 score, and Brier score were calculated with R “caret,” “epiR,” and “rms” packages. Kaplan-Meier plot with log-rank test was conducted with R “survival” and “survminer” packages. Model importance was calculated using R package “caret.” The correlation between selected features and critical illness status were calculated Spearman correlation. Significance of the difference between the median values of critical illness and non-critical illness were conducted by the Asymptotic Two-Sample Brown-Mood Median Test using R “coin” package. *P* values less than 0.05 were considered statistically significant. Univariate and multivariate Cox regression were conducted with R “survival” package. Ninety-five percent confidence interval (CI) are reported if necessary.

## Results

### Baseline characteristics of patients

A total of 450 patients were finally included in this study, with 111 patients in the training cohort, 111 patients in the internal validation cohort, and 228 patients in external validation cohort. Median age in the training cohort, internal validation cohort, and external validation cohort was 62 (54.5–72) years, 64 (52–70.5) years, and 63 (50-70) years, respectively. Common comorbidities, such as hypertension and diabetes, and major symptoms of COVID-19, including fever, cough, dyspnea, sputum, and fatigue, were similar among the three cohorts. The number of critically ill patients in these three cohorts was 12 (10.81%), 16 (14.41%), and 25 (10.96%) in turn. Detailed demographic and essential clinical characteristics are listed in Table 1.

**Table 1:** Baseline characteristics of individuals by cohorts

Characteristics	Training cohort	Internal validation cohort	External validation cohort
	(***N*** = 111)	(***N*** = 111)	(***N*** = 228)
**Demographics**			
Age, years	62 (54.5-72)	64 (52-70.5)	63 (50-70)
Sex			
Female	65 (58.56%)	53 (47.75%)	116 (50.88%)
Male	46 (41.44%)	58 (52.25%)	112 (49.12%)
**Clinical characteristics**			
Comorbidity number	1 (0-2)	1 (1-2)	1 (0-2)
Comorbidity			
Hypertension	40 (36.04%)	52 (46.85%)	86 (37.72%)
Diabetes	21 (18.92%)	26 (23.42%)	41 (17.98%)
Coronary heart disease	6 (5.41%)	12 (10.81%)	16 (7.02%)
Tumor	8 (7.21%)	12 (10.81%)	17 (7.46%)
COPD	1 (0.9%)	2 (1.8%)	2 (0.88%)
**Symptoms at admission**			
Fever	97 (87.39%)	90 (81.08%)	166 (72.81%)
Temp (max) ≥ 39 °C	28 (25.23%)	24 (21.62%)	45 (19.74%)
Cough	78 (70.27%)	79 (71.17%)	170 (74.56%)
Dyspnea	56 (50.45%)	51 (45.95%)	88 (38.6%)
Sputum	44 (39.64%)	49 (44.14%)	108 (47.37%)
Fatigue	48 (43.24%)	49 (44.14%)	56 (24.56%)
Diarrhea	31 (27.93%)	34 (30.63%)	35 (15.35%)
Myalgia	23 (20.72%)	36 (32.43%)	34 (14.91%)
Nausea or vomiting	3 (2.7%)	7 (6.31%)	2 (0.88%)
**Vital status**			
Critical illness	12 (10.81%)	16 (14.41%)	25 (10.96%)
Noncritical illness	99 (89.19%)	95 (85.59%)	203 (89.04%)
**Follow-up**			
Time to critical illness, days	25 (14.5-34)	25 (10-30.5)	18.5 (11.75-30.25)

Continuous variables were presented as median (interquartile ranges [IQR]) while categorical variables as counts and percentages (%)

*COPD*, chronic obstructive pulmonary disease

### Features included in models

**Fig. 1:**
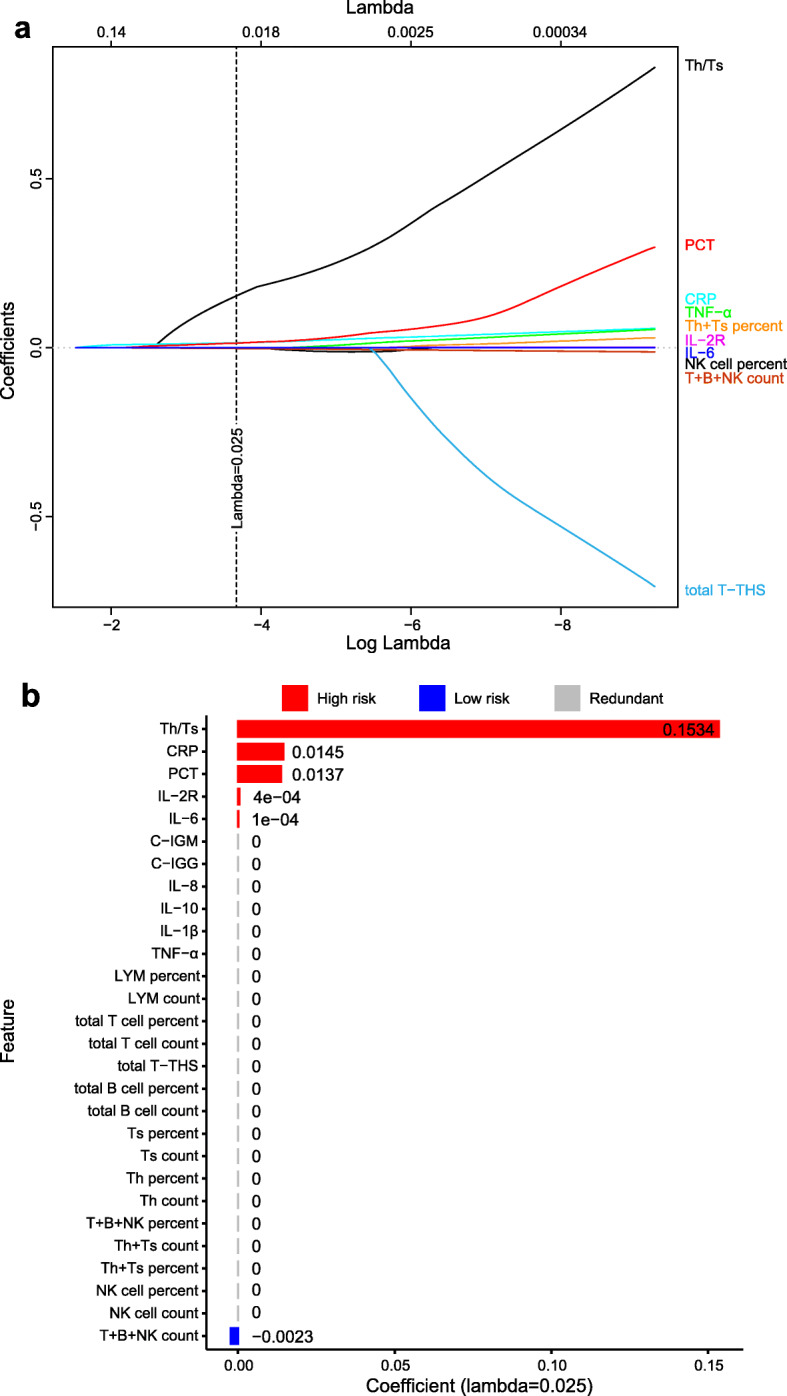
Feature selection by LASSO. **a** LASSO variable trace profiles of the 6 features. The vertical dashed line shows the best lambda value (0.025) chosen by tenfold cross validation. **b** Feature with zero coefficient (colored with gray) at lambda = 0.025, was considered less crucial to the patient’s critical illness status and removed by Lasso logistic regression analysis. Feature with positive coefficient (colored with red) are regarded high risk in respect to critical illness. LASSO, least absolute shrinkage and selection operator. CRP, C reactive protein. PCT, procalcitonin. IL-2R, interleukin 2 receptor. IL-6, interleukin 6. T + B + NK, T lymphocyte and B lymphocyte and natural killer cells. Th/Ts,T-helper/T-suppressor lymphocyte

After feature filtering, 28 features were left for feature selection, including NK cell count, NK cell percent, (Th + Ts) percent, Th/Ts, (Th + Ts) count, (T + B + NK) count, (T + B + NK) percent, Th count, Th percent, Ts count, Ts percent, total B count, total B percent, total T-THS, total T count, total T percent, LYM count, LYM percent, CRP, PCT, TNF-α, IL-1β, IL-2R, IL-6, IL-10, IL-8, C-IGG, and C-IGM (Fig. 1a). Missing feature value imputation was then conducted utilizing random forest. LASSO logistic regression identified six features (Th/Ts, CRP, PCT, IL-2R, IL-6, [T + B + NK] count) with the most predictive performance for model development. Among these features, (T + B + NK) count was negatively correlated with critical illness (–0.0023), while the other five features, Th/Ts (0.1534), CRP (0.0145), PCT (0.0137), IL-2R (4e − 04), and IL-6 (1e − 04), were positively correlated with critical illness (Fig. 1b).

**Fig. 2:**
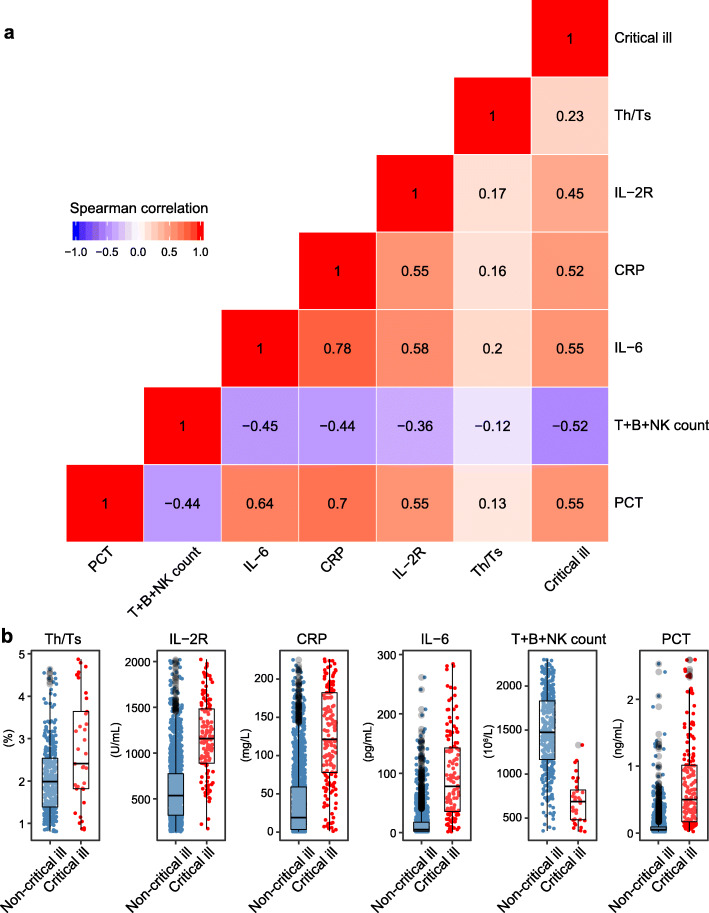
Statistical analysis of six features selected by Lasso. **a** Spearman correlation of critical illness status and features. The wider chord, the stronger positive correlation is. **b** Density plot of each feature across patients with different critical illness status, respectively. The vertical dashed line signifies the feature median value, interquartile range is also annotated. The significant test is Asymptotic Two-Sample Brown-Mood Median Test. CRP, C reactive protein. PCT, procalcitonin. IL-2R, interleukin 2 receptor. IL-6, interleukin 6. T + B + NK, T lymphocyte and B lymphocyte and natural killer cells. Th/Ts,T-helper/T-suppressor lymphocyte

As shown in Fig. 2a, we conducted the Spearman correlation analysis between the six features and critical illness status, the results of which were consistent with that of LASSO analysis. The five unfavorable prognostic features identified by LASSO were positively correlated with critical illness at varying degrees. The top-weighted features IL-6 (*R* = 0.55), PCT (*R* = 0.55), CRP (*R* = 0.52), IL-2R (*R* = 0.45), and Th/Ts (*R* = 0.23) were consistent with previously reported risk factors intimately associated with poor outcome of COVID-19 [4–6, 22, 23].

Significant differences (*p* < 0.05) of the six features between critically ill and non-critically ill patients with COVID-19 were presented in the standard box plots (Fig. 2b). The values of Th/Ts ratio, IL-2R, CRP, IL-6, and PCT, were significantly higher in critically ill patients than that in non-critically ill group, while (T + B + NK) count was lower in critically ill patients (Additional file 1).

### Model performance

**Fig. 3:**
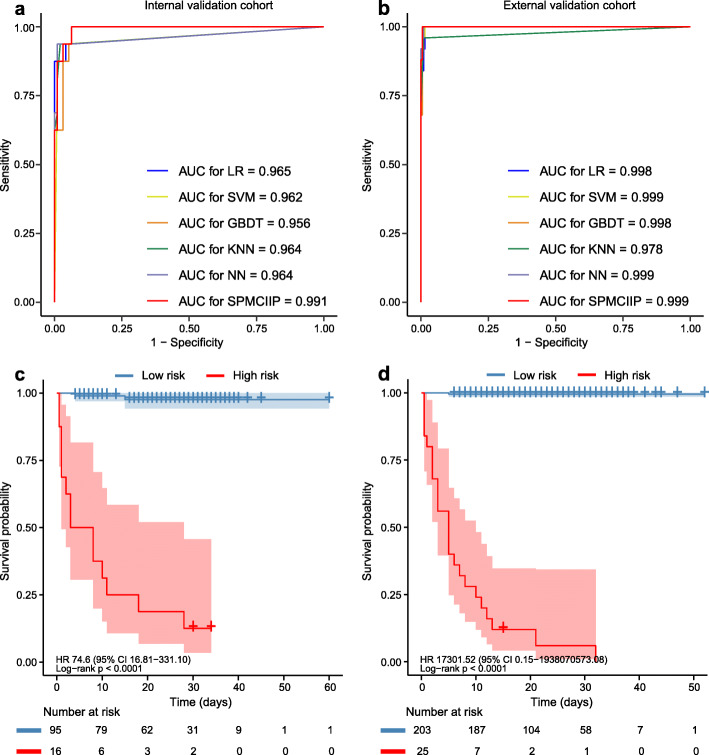
Performance evaluation on the validation dataset. **a**, **b** ROC curve and AUC of SVM, LR, GBDT, KNN, and NN in internal validation cohort and external validation cohort, respectively. **c**, **d** KM curve of low-risk and high-risk subgroup predicted by SVM model in internal validation cohort and external validation cohort, respectively. The light red or blue areas refer to the 95% confidence interval. *p* value is computed by log-rank test. Hazard ratio (HR) and its 95% confidence interval are obtained with univariate Cox model. SVM, supported vector machine. LR, logistic regression. GBDT, gradient boosted decision tree. KNN, k-nearest neighbor. NN, neural network. HR, hazard ratio

In general, all five models (LR, SVM, GBDT, KNN, and NN) showed varying but promising critical illness risk prediction performance across cohorts. The AUC was 0.965 with LR, 0.962 with SVM, 0.956 with GBDT, 0.964 with KNN, and 0.964 with NN for the internal validation cohort (Fig. 3a). The AUC was 0.998 with LR, 0.999 with SVM, 0.998 with GBDT, 0.978 with KNN, and 0.999 with NN for the external validation cohort (Fig. 3b). Among them, the ensemble model SPMCIIP (severity prediction model for COVID-19 by immune-inflammatory parameters) derived from three algorithms (SVM, GBDT, and NN) achieved the best predictive performance. Relative importance of features included in SPMCIIP and its baseline models is shown in Additional file 4.

For the internal validation cohort, SPMCIIP achieved an AUC of 0.991 (95% CI 0.979–1.000) to identify patients having a high risk of developing critical illness with an accuracy of 96.4% (95% CI 91.0%–99.0%). For external validation cohort, SPMCIIP demonstrated an AUC of 0.999 (95% CI 0.998–1.000) and an accuracy of 99.1% (95% CI 96.9%–99.9%). The calibration curve of SPMCIIP in two validation cohorts is depicted in Additional file 5, showing that SPMCIIP also displayed the minimal Brier score of 0.025 for internal validation cohort and 0.007 for external validation cohort. All other metrics and the performance of the baseline models are listed in Table 2.

**Table 2:** Performance metrics for mortality risk prediction of models in cohorts

	AUC (95% CI)	Accuracy (95% CI)	SN (95% CI)	SP (95% CI)	PPV (95% CI)	NPV (95% CI)	Kappa	F1	Brier
**Internal validation cohort**									
**LR**	0.965 (0.9-1)	0.982 (0.936-0.998)	0.875 (0.617-0.985)	1 (0.962-1)	1 (0.768-1)	0.979 (0.928-0.998)	0.923	0.933	0.018
**SVM**	0.962 (0.898-1)	0.982 (0.936-0.998)	0.938 (0.698-0.998)	0.990 (0.943-1)	0.938 (0.698-0.998)	0.990 (0.943-1)	0.927	0.938	0.018
**GBDT**	0.956 (0.891-1)	0.955 (0.898-0.985)	0.875 (0.617-0.985)	0.968 (0.911-0.993)	0.824 (0.566-0.962)	0.979 (0.925-0.997)	0.822	0.849	0.045
**KNN**	0.964 (0.899-1)	0.973 (0.923-0.994)	0.938 (0.698-0.998)	0.979 (0.926-0.997)	0.882 (0.636-0.985)	0.989 (0.942-1)	0.893	0.909	0.024
**NN**	0.964 (0.899-1)	0.973 (0.923-0.994)	0.938 (0.698-0.998)	0.979 (0.926-0.997)	0.882 (0.636-0.985)	0.989 (0.942-1)	0.893	0.909	0.027
**SPMCIIP**	0.991 (0.979-1)	0.964 (0.910-0.990)	0.875 (0.617-0.985)	0.979 (0.926-0.997)	0.875 (0.617-0.985)	0.979 (0.926-0.997)	0.854	0.875	0.025
**External validation cohort**									
**LR**	0.998 (0.995-1)	0.978 (0.950-0.993)	0.8 (0.593-0.932)	1 (0.982-1)	1 (0.832-1)	0.976 (0.945-0.992)	0.877	0.889	0.022
**SVM**	0.999 (0.998-1)	0.987 (0.962-0.997)	0.92 (0.740-0.990)	0.995 (0.973-1)	0.958 (0.789-0.999)	0.990 (0.965-0.999)	0.931	0.939	0.011
**GBDT**	0.998 (0.995-1)	0.987 (0.962-0.997)	0.96 (0.797-0.999)	0.990 (0.965-0.999)	0.923 (0.749-0.991)	0.995 (0.973-1)	0.934	0.941	0.013
**KNN**	0.978 (0.939-1)	0.987 (0.962-0.997)	0.96 (0.797-0.999)	0.990 (0.965-0.999)	0.923 (0.749-0.991)	0.995 (0.973-1)	0.934	0.941	0.012
**NN**	0.999 (0.998-1)	0.991 (0.969-0.999)	0.96 (0.797-0.999)	0.995 (0.973-1)	0.96 (0.797-0.999)	0.995 (0.973-1)	0.955	0.960	0.009
**SPMCIIP**	0.999 (0.998-1)	0.991 (0.969-0.999)	0.96 (0.797-0.999)	0.995 (0.973-1)	0.96 (0.797-0.999)	0.995 (0.973-1)	0.955	0.960	0.007

*LR* logistic regression, *SVM* supported vector machine, *GBDT* gradient boosted decision tree, *KNN* k-nearest neighbor, *NN* neural network, *SPMCIIP* severity prediction model for COVID-19 by immune-inflammatory parameters, *AUC* area under the curve, *SN* sensitivity, *SP* specificity, *PPV* positive predictive value, *NPV* negative predictive value, *CI* confidence interval

Taking critical illness as endpoint and time from admission to occurrence of critical COVID-19 or discharge as the endpoint, Kaplan-Meier analysis further confirmed the strong risk stratification ability of SPMCIIP. SPMCIIP robustly stratified high-risk patients and low-risk patients with *p* < 0.0001 in both internal and external validation cohorts. The univariate Cox analysis also demonstrated the strong positive correlation between SPMCIIP predicted critical illness subgroup and the ground truth critical illness survival for internal (HR, 74.6, 95% CI 16.81–331.10) and external (HR, 17,301.52, 95% CI 0.15–1,938,070,573.08) validation cohorts, respectively (Fig. 3c, d).

**Fig. 4:**
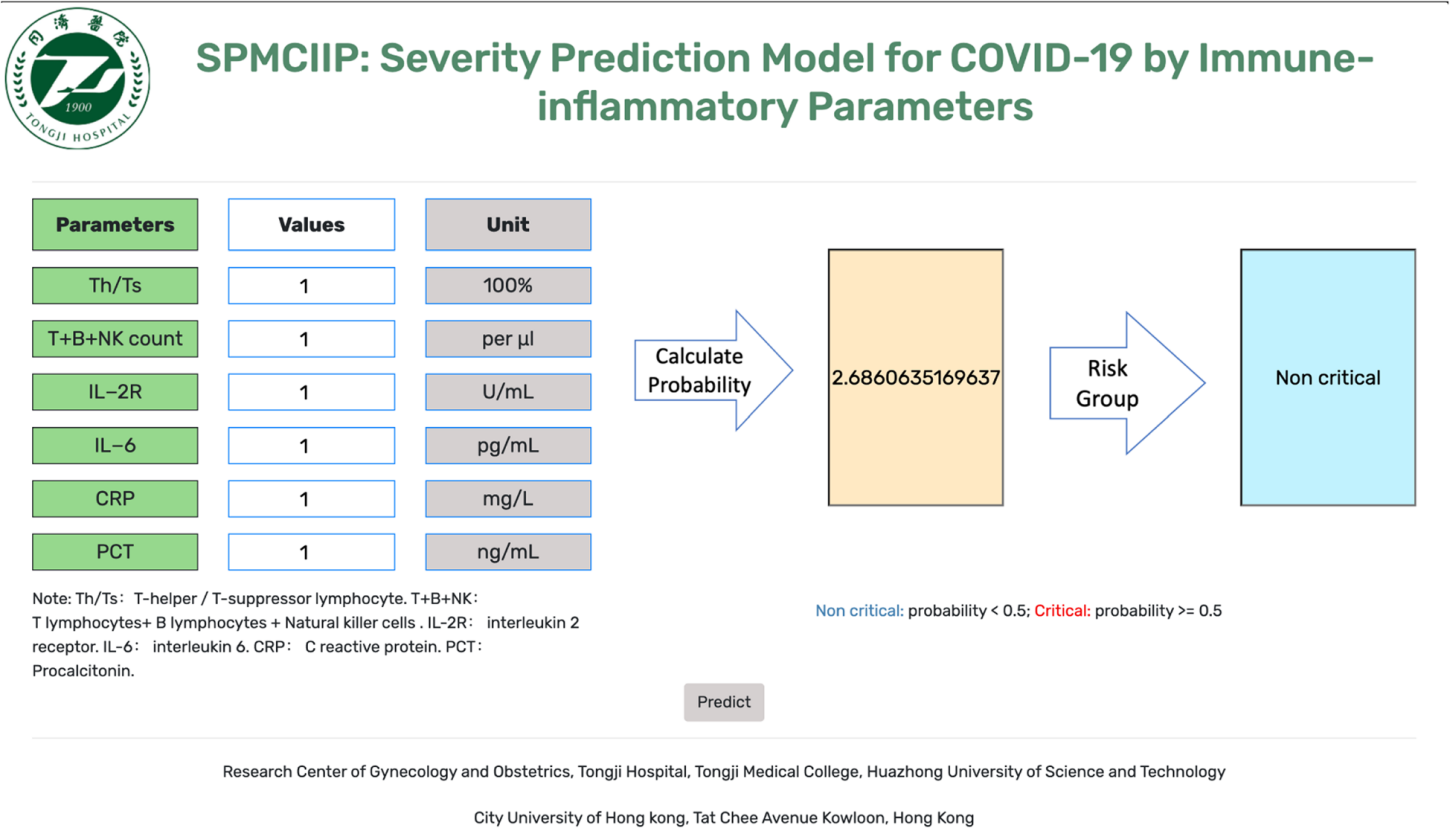
Illustration of the online prediction model—SPMCIIP

We also developed an online calculator where directly inputting the values of parameters could yield the risk of developing critical COVID-19 (https://spmciip.deepomics.org/). After the clinicians fill in the online form with corresponding features, SPMCIIP returns a personalized probability and risk group of critical illness. Illustration of an example of the online prediction system is presented in Fig. 4.

## Discussion

In this study, we developed and validated an ensemble machine learning model based on immune-inflammatory parameters to predict the risk of critical COVID-19. We conducted and reported this multicenter retrospective study following appropriate standards [24]. Importantly, SPMCIIP displayed an AUC exceeding 0.99 to accurately predict critical COVID-19 in both internal and external validation cohorts. With an expeditious risk stratification of patients’ prognosis, clinicians can strengthen the management of patients at high risk of critical illness, which assists to curb mortality and rationally allocate medical resources.

The six features involved in SPMCIIP had been proven correlated with critical illness in COVID-19 patients. Severity of COVID-19 is due to the viral infection and the host response, and critical COVID-19 is a distinct clinical and immune sepsis subphenotype [25]. Innate immune hyperactivation and adaptive immune dysregulation after SARS-CoV-2 infection are considered to play important roles in the development of severe COVID-19 [26]. The vast release of cytokines in response to the viral infection can result in a cytokine storm and symptoms of sepsis. Uncontrolled inflammation inflicts multi-organ damage, leads to multi-organ failure including acute respiratory distress syndrome, and finally results in poor prognosis of COVID-19 [8, 27, 28]. The increase of inflammatory factors and cytokines was observed, especially in critically ill patients. High level of IL-6 was early reported to be correlated with SARS-CoV-2 viral load in the blood of critically ill COVID-19 patients [29]. IL-6 can end the activation of normal T cells, which may be a reason for lymphopenia; robust proinflammatory function; and inducing a variety of acute-phase proteins, such as CRP. It is even reported that the immune dysregulation is driven by IL-6 in COVID-19 [30]. With a median incubation time of 5–7 days, and 3–4 days additionally from hospitalization to requirement of mechanical ventilation or admission to ICU [31], this subacute pattern of progression in COVID-19 patients raises the possibility of immunosuppression, due both to T cell depletion and exhaustion after over-activation [26, 32]. Consistent with it, CD4+ T, CD8+ T, and NK cells were observed lower in patients with severe disease [33]. Corresponding to this finding, single-cell sequencing of peripheral blood mononuclear cells reveals that the expression of multiple genes related to apoptosis pathway was upregulated in T, B, NK cell subsets of COVID-19 patients comparing with healthy people [34]. Lymphopenia, especially the depletion of T cells, may relate to apoptosis following overactive inflammatory responses. Further, CD4+ T cell and NK cell cytopenia are recognized as characteristics of infection by SARS-CoV-2 [30]. In addition, procalcitonin is correlated with increased probability of bacterial pathogens [35], and several studies have demonstrated that higher procalcitonin was presented in critically ill COVID-19 patients [6, 36, 37]. This finding indicates bacterial co-infection in critically ill patients. More accurately, the prevalence of bacterial co-infection in critically ill COVID-19 patients (14%, 95% CI 5-26) in ICU is higher than that in hospitalized COVID-19 patients (7%, 95% CI 3-12%), according to a recent meta-analysis [38].

Though the process of COVID-19 infection has not been fully clarified, the driving role of immune dysfunction on critical COVID-19 is becoming more evident, fueling us to leverage immunological features in predicting critical illness. Machine learning can help clinicians predict the health trajectory of patients, and aid preventative efforts for improving outcomes [39]. Besides, machine learning models could predict disregarding human fatigue, geographic barriers, and temporal restrictions in an automated manner. Therefore, a machine learning model based on immune-inflammatory parameters could offer great opportunities to accurate prediction of critical COVID-19 when medical resources are scarce and COVID-19 infections surge.

Importantly, SPMCIIP can predict the risk of progressing to critical COVID-19 nearly 20 days in advance. Because the impacts of cytokine release syndrome caused by SARS-CoV-2 infection on COVID-19 have been increasingly revealed, and understandings of the use of corticosteroids and other anti-inflammatory drugs continue to grow [40, 41], early identification of patients harboring high risk of critical illness potentially facilitates timely intervention in compliance with guidelines and eliminate the occurrence of cytokine storm-derived multiorgan failure and other refractory states.

The merits of SPMCIIP include its excellent performance in predicting critical COVID-19. Many machine learning models for prognosis prediction of COVID-19 have been built based on imaging and clinical features [16, 42], but few models could yield an AUC as high as 99% to predict critical COVID-19. In the case of limited medical conditions, such as clinics and small hospitals, a prediction model with parameters easily determined is appropriate, once medical conditions permit, the six features included in the model are able to be detected, it is recommended to use the online model SPMCIIP. The predictive advantage of SPMCIIP may attribute to the algorithms we adopted, which covered most types of classification models in machine learning and enabled dealing with complex data. More importantly, the predictive superiority of SPMCIP is owing to the immune and inflammatory features used for model development. While myriad risk factors associated with occurrence of critical COVID-19 have been unveiled, it is gradually recognized that the interplay between immunity and inflammation is the predominant factor that affects the outcome of COVID-19 [43–45]. Our results further demonstrated the heterogeneity of immune response in COVID-19 patients and its important prognostic value delineated previously [46, 47]. The predictive strength of SPMCIIP could stem from the detailed feature information of included patients, though the number of eligible patients is relatively limited (450/2076).

Our research has some limitations. First, patients included in this study are primarily locals in Wuhan, China. Validations of SPMCIIP in other regions and ethnicities can provide more solid evidence. Second, this is a retrospective study. Our models should be independently validated in large-scale prospective cohorts before the contribution to improved survival can be elucidated.

## Conclusions

In this multicenter retrospective study, we developed and validated an online model, SPMCIIP, which included six immune and inflammatory parameters and could accurately predict the critical illness risk of COVID-19 patients, thus triaging patients for appropriate treatment and optimizing the use of medical resources.

## Supplementary Information

**Additional file 1 **Differential variables between critical ill and non-critical ill patients. The significant test is Asymptotic Two-Sample Brown-Mood Median Test. *Abbreviations:* Th/Ts, T-helper/T-suppressor lymphocyte. IL-2R, interleukin 2 receptor. CRP, C reactive protein. IQR, interquartile ranges.

**Additional file 2.** Visualization of the denosing and filtering process. a, Heatmap of raw lab test data. b, Heatmap of lab test data after removing patients with more than and equal to 30% missing entries across the SF and OV hospitals. c, Heatmap of lab test data after removing lab test features with more than and equal to 30% missing entries across the SF and OV hospitals. Black tiles refer to missing entries. Abbreviations: NK, Natural killer cells, Th, T-helper lymphocyte. Ts, T-suppressor lymphocyte. C3, complement 3. C4, complement 4. CRP, C reactive protein. PCT, procalcitonin IFN-γ, interferon-γ. TNF-α, tumor necrosis factor α. IL-1β, interleukin 1β. IL-2R, interleukin 2 receptor. IL-4, interleukin 4. IL-6, interleukin 6. IL-8, interleukin 8. IL-10, interleukin 10. IGA, immunoglobulin A. IGG, immunoglobulin G. IGM, immunoglobulin M. C-IGM, SARS-COV-2 specific antibody IgM. C-IGG, SARS-COV-2 specific antibody IgG. SF, Sino-French New City Campus of Tongji Hospital. OV, Optical Valley Campus of Tongji Hospital.

**Additional file 3 **Visualization of the imputation process. **a, c** Heatmap of SF and OV lab test data before imputation. **b, d** Heatmap of SF and OV lab test data after imputation. Black tiles refer to missing entries. *Abbreviations*: NK, Natural killer cells, Th, T-helper lymphocyte. Ts, T-suppressor lymphocyte. CRP, C reactive protein. PCT, procalcitonin. IFN-γ, interferon-γ. TNF-α, tumor necrosis factor α. IL-1β, interleukin 1β. IL-2R, interleukin 2 receptor. IL-4, interleukin 4. IL-6, interleukin 6. IL-8, interleukin 8. IL-10, interleukin 10. C-IGM, SARS-COV-2 specific antibody IgM. C-IGG, SARS-COV-2 specific antibody IgG. SF, Sino-French New City Campus of Tongji Hospital. OV, Optical Valley Campus of Tongji Hospital.

**Additional file 4 **Relative feature importance of SVM, GBDT, NN and SPMCIIP model. *Abbreviations*: SVM, supported vector machine. GBDT, Gradient Boosted Decision Tree. NN, neural network. SPMCIIP, Severity prediction model for COVID-19 by immune-inflammatory parameters. CRP, C reactive protein. IL-2R, interleukin 2 receptor. IL-6, interleukin 6. NK, Natural killer cells. PCT, procalcitonin. Th, T-helper lymphocyte. Ts, T-suppressor lymphocyte.

**Additional file 5 **Calibration curves of SPMCIIP model in cohorts. Calibration curves of SPMCIIP model in **a** internal validation cohort and **b** external validation cohort, respectively. The triangle represents the observation group. Each group contained an average of 20 observations. The dashed line is the ideal calibration curve. The bottom vertical lines refer to the predicted probability distribution. Red curve is the fitted nonparametric calibration curve. *Abbreviations*: AUC, Area under the curve.

## Data Availability

The datasets generated and analyzed during the current study are available from the corresponding author upon reasonable request.

## Abbreviations

ACC: Accuracy
AUC: Area under the curve
C3: Complement 3
C4: Complement 4
CI: Confidence interval
C-IgG: SARS-CoV-2 specific antibody IgG
C-IgM: SARS-CoV-2 specific antibody IgM
COVID-19: Coronavirus disease 2019
GBDT: Gradient Boosted Decision Tree
CRP: C reactive protein
IFN-γ: Interferon-γ
IgA: Immunoglobulin A
IgG: Immunoglobulin G
IgM: Immunoglobulin M
IL-10: Interleukin 10
IL-1β: Interleukin 1β
IL-2R: Interleukin 2 receptor
IL-4: Interleukin 4
IL-6: Interleukin 6
IL-8: Interleukin 8
KNN: K-Nearest Neighbor
LASSO: Least Absolute Shrinkage and Selection Operator
LR: Logistic Regression
LYM: Lymphocyte
NK cells: Natural killer cells
NN: Neural Network
NPV: Negative predictive value
PCT: Procalcitonin
PPV: Positive predictive value
ROC: Receiver operating characteristics
SARS-CoV-2: Severe acute respiratory syndrome coronavirus 2
SE: Sensitivity
SN: Sensitivity
SP: Specificity
SPMCIIP: A severity prediction model for COVID-19 by immune-inflammatory parameters
SVM: Support Vector Machine
Th: T-helper lymphocyte
Ts: T-suppressor lymphocyte
TNF-α: Tumor necrosis factor α
