# Supplementary material for: Development and validation of an online model to predict critical COVID-19 with immune-inflammatory parameters
Source: J Intensive Care. 2021 Feb 18;9:19. doi: 10.1186/s40560-021-00531-1 (PMC7891473; doi:10.1186/s40560-021-00531-1)
Supplement: Supplementary file 1 — Additional file 1 Differential variables between critical ill and non-critical ill patients. The significant test is Asymptotic Two-Sample Brown-Mood Median Test. Abbreviations: Th/Ts, T-helper/T-suppressor lymphocyte. IL-2R, interleukin 2 receptor. CRP, C reactive protein. IQR, interquartile ranges. [file 40560_2021_531_MOESM1_ESM.docx]

**Differential variables between critical ill and non-critical ill patients.**

| Features | Critical ill | Non-critical ill | *p* value^a^ |
| --- | --- | --- | --- |
| Th/Ts (median [IQR]) | 3.29 [1.85, 5.01] | 1.98 [1.32, 2.54] | 0.006 |
| IL-2R (median [IQR]),U/ml | 1447.0 [993.0, 2327.5] | 520.0 [299.0, 770.5] | <0.001 |
| CRP (median [IQR]),mg/l | 182.7 [103.2, 258.6] | 16.1 [2.5, 57.8] | <0.001 |
| IL-6 (median [IQR]),pg/ml | 169.40 [58.14, 640.90] | 5.01 [2.07, 18.39] | <0.001 |
| (T+B+NK) count (median [IQR]), per ul | 424.0 [263.75, 718.5] | 1517.5 [1184.8, 1884.0] | <0.001 |
| PCT (median [IQR]),ng/ml | 1.02 [0.34, 4.45] | 0.06 [0.04, 0.09] | <0.001 |

^a^The significant test is Asymptotic Two-Sample Brown-Mood Median Test.

Th/Ts, T-helper/T-suppressor lymphocyte. IL-2R, interleukin 2 receptor. CRP, C reactive protein. IL-6: interleukin 6. T+B+NK: T lymphocyte and B lymphocyte and Natural killer cells. PCT: procalcitonin. IQR: interquartile ranges
