# Supplementary figures and images for: Development and validation of an online model to predict critical COVID-19 with immune-inflammatory parameters
Source: J Intensive Care. 2021 Feb 18;9:19. doi: 10.1186/s40560-021-00531-1 (PMC7891473; doi:10.1186/s40560-021-00531-1)

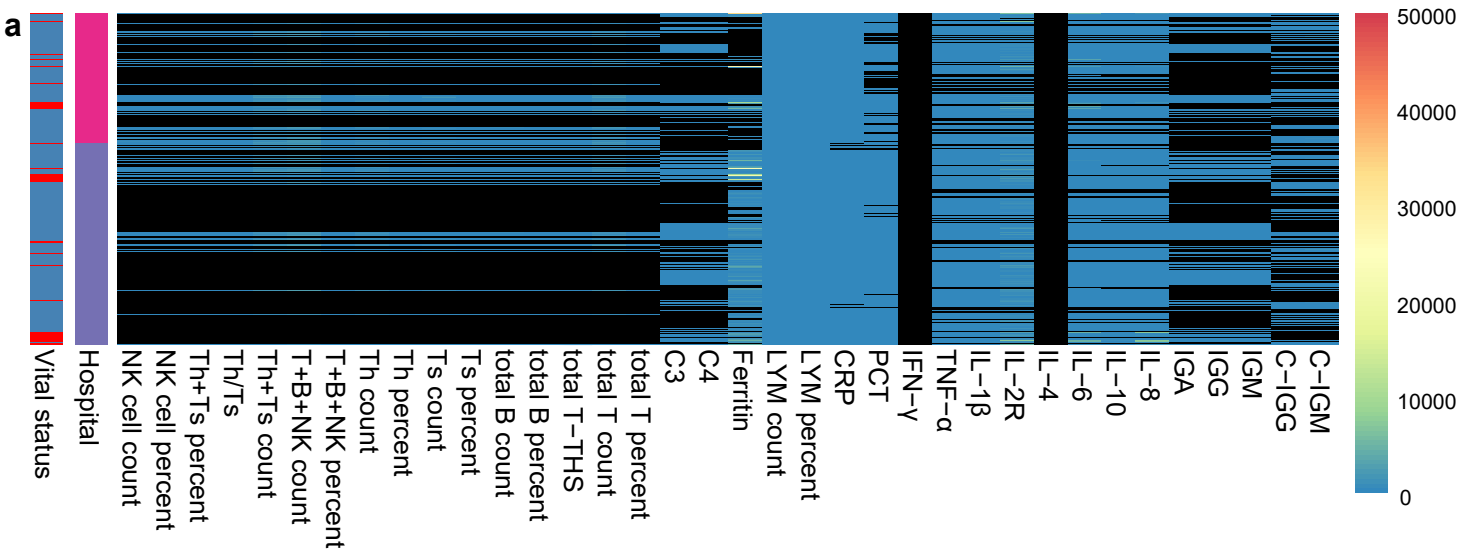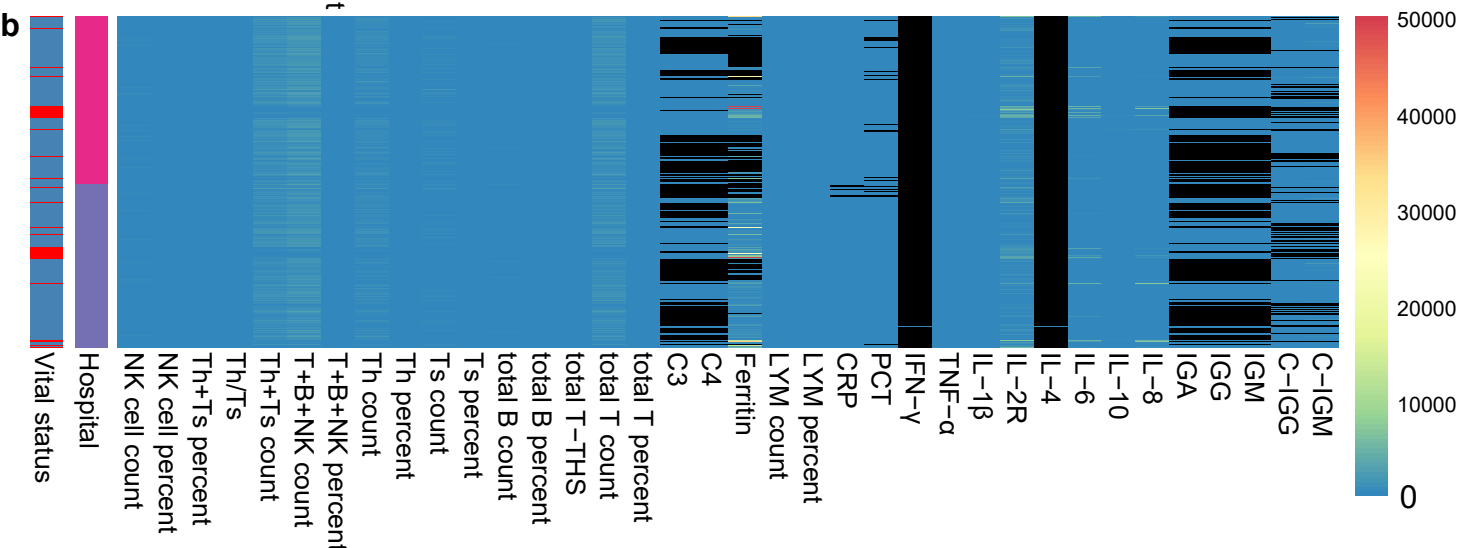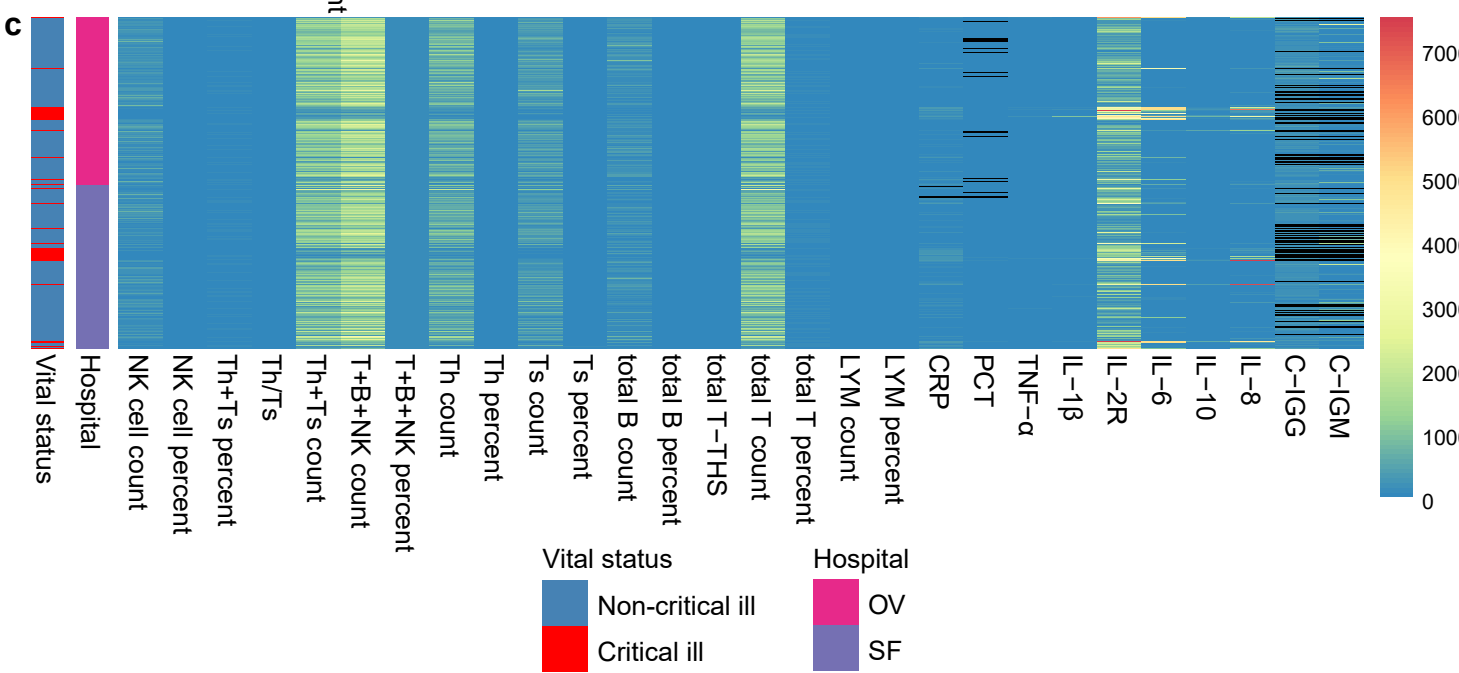

Supplement: Supplementary file 2 — Additional file 2. Visualization of the denosing and filtering process. a, Heatmap of raw lab test data. b, Heatmap of lab test data after removing patients with more than and equal to 30% missing entries across the SF and OV hospitals. c, Heatmap of lab test data after removing lab test features with more than and equal to 30% missing entries across the SF and OV hospitals. Black tiles refer to missing entries. Abbreviations: NK, Natural killer cells, Th, T-helper lymphocyte. Ts, T-suppressor lymphocyte. C3, complement 3. C4, complement 4. CRP, C reactive protein. PCT, procalcitonin IFN-γ, interferon-γ. TNF-α, tumor necrosis factor α. IL-1β, interleukin 1β. IL-2R, interleukin 2 receptor. IL-4, interleukin 4. IL-6, interleukin 6. IL-8, interleukin 8. IL-10, interleukin 10. IGA, immunoglobulin A. IGG, immunoglobulin G. IGM, immunoglobulin M. C-IGM, SARS-COV-2 specific antibody IgM. C-IGG, SARS-COV-2 specific antibody IgG. SF, Sino-French New City Campus of Tongji Hospital. OV, Optical Valley Campus of Tongji Hospital. [file 40560_2021_531_MOESM2_ESM.pdf]

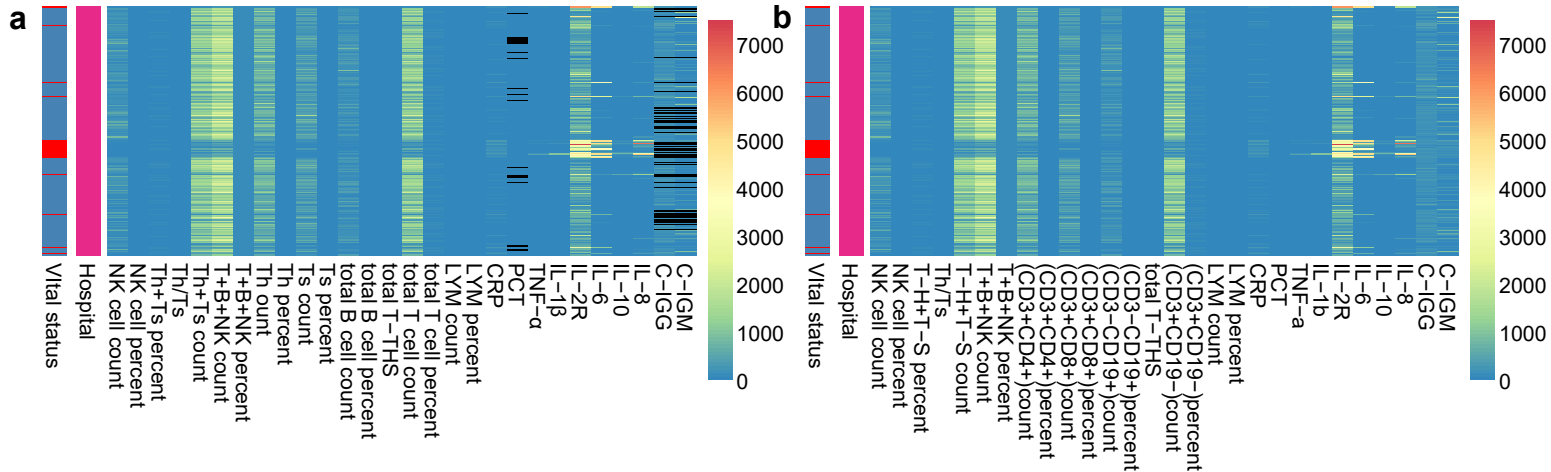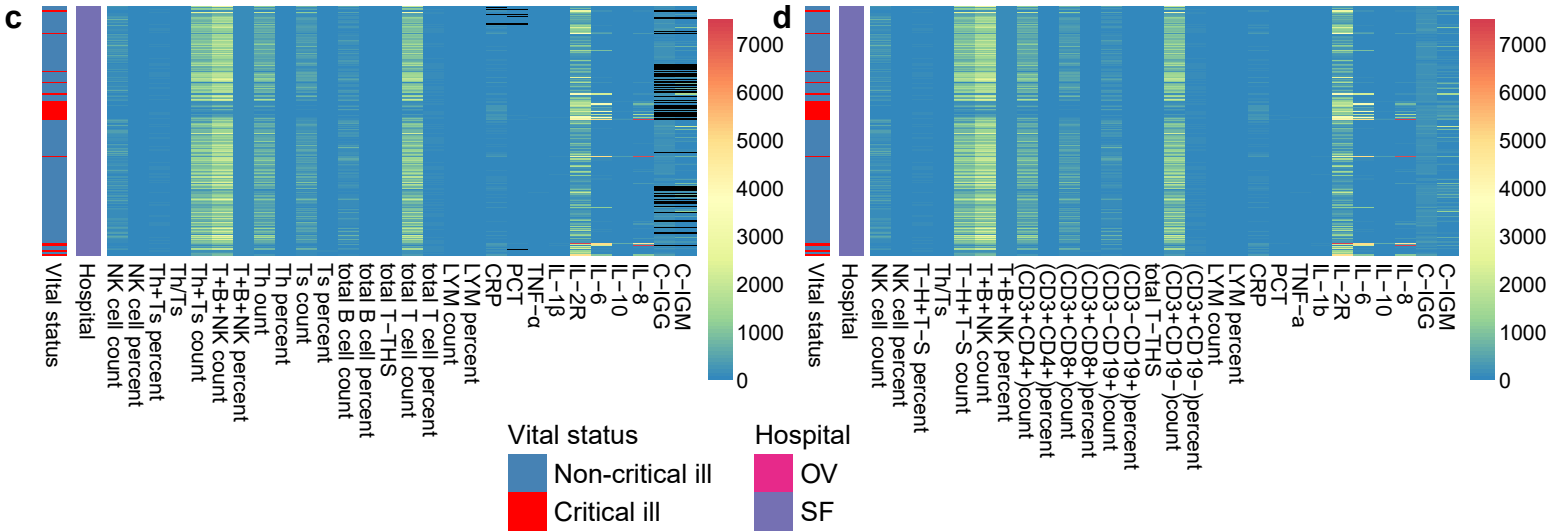

Supplement: Supplementary file 3 — Additional file 3 Visualization of the imputation process. a, c Heatmap of SF and OV lab test data before imputation. b, d Heatmap of SF and OV lab test data after imputation. Black tiles refer to missing entries. Abbreviations: NK, Natural killer cells, Th, T-helper lymphocyte. Ts, T-suppressor lymphocyte. CRP, C reactive protein. PCT, procalcitonin. IFN-γ, interferon-γ. TNF-α, tumor necrosis factor α. IL-1β, interleukin 1β. IL-2R, interleukin 2 receptor. IL-4, interleukin 4. IL-6, interleukin 6. IL-8, interleukin 8. IL-10, interleukin 10. C-IGM, SARS-COV-2 specific antibody IgM. C-IGG, SARS-COV-2 specific antibody IgG. SF, Sino-French New City Campus of Tongji Hospital. OV, Optical Valley Campus of Tongji Hospital. [file 40560_2021_531_MOESM3_ESM.pdf]

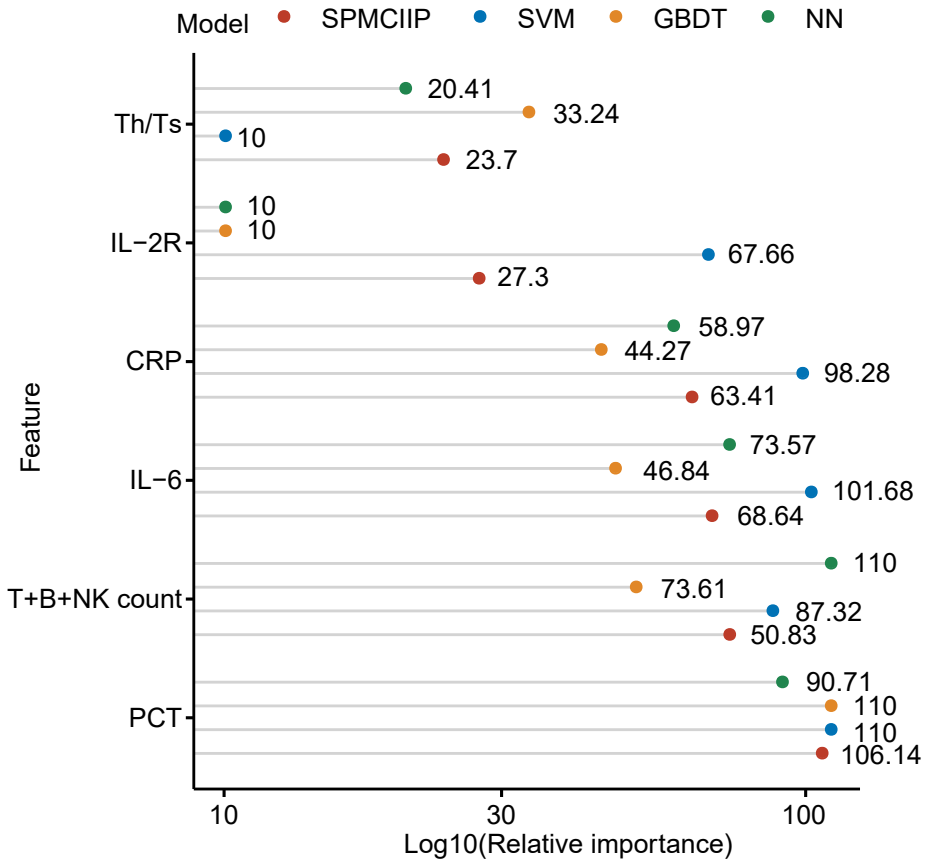

Supplement: Supplementary file 4 — Additional file 4 Relative feature importance of SVM, GBDT, NN and SPMCIIP model. Abbreviations: SVM, supported vector machine. GBDT, Gradient Boosted Decision Tree. NN, neural network. SPMCIIP, Severity prediction model for COVID-19 by immune-inflammatory parameters. CRP, C reactive protein. IL-2R, interleukin 2 receptor. IL-6, interleukin 6. NK, Natural killer cells. PCT, procalcitonin. Th, T-helper lymphocyte. Ts, T-suppressor lymphocyte. [file 40560_2021_531_MOESM4_ESM.pdf]

Internal validation cohort

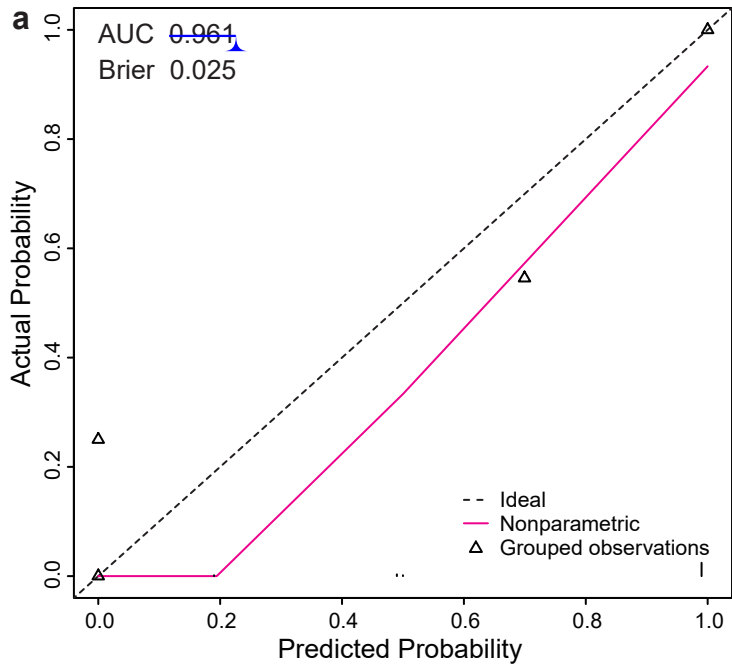

External validation cohort

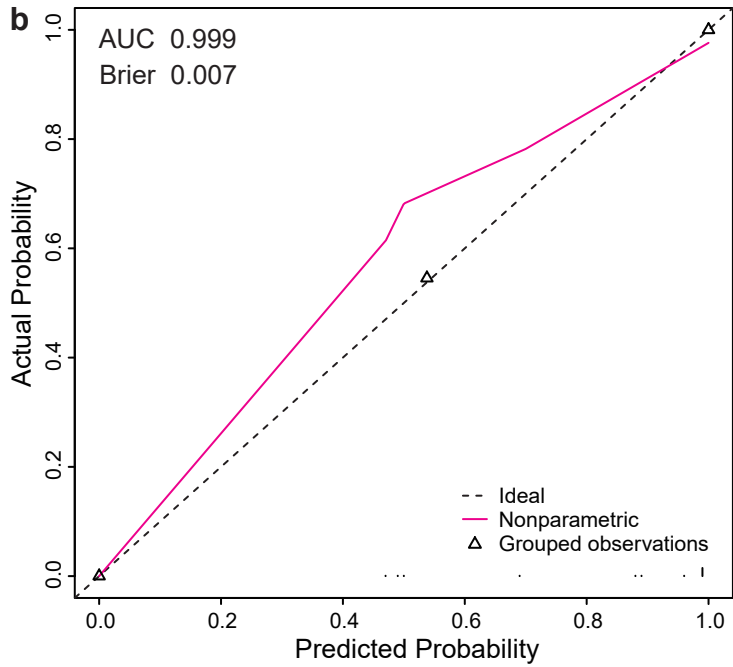

Supplement: Supplementary file 5 — Additional file 5 Calibration curves of SPMCIIP model in cohorts. Calibration curves of SPMCIIP model in a internal validation cohort and b external validation cohort, respectively. The triangle represents the observation group. Each group contained an average of 20 observations. The dashed line is the ideal calibration curve. The bottom vertical lines refer to the predicted probability distribution. Red curve is the fitted nonparametric calibration curve. Abbreviations: AUC, Area under the curve. [file 40560_2021_531_MOESM5_ESM.pdf]
